# Predicting Cellular Growth from Gene Expression Signatures

**DOI:** 10.1371/journal.pcbi.1000257

**Published:** 2009-01-02

**Authors:** Edoardo M. Airoldi, Curtis Huttenhower, David Gresham, Charles Lu, Amy A. Caudy, Maitreya J. Dunham, James R. Broach, David Botstein, Olga G. Troyanskaya

**Affiliations:** 1 Lewis-Sigler Institute for Integrative Genomics, Carl Icahn Laboratory, Princeton University, Princeton, New Jersey, United States of America; 2 Department of Computer Science, Princeton University, Princeton, New Jersey, United States of America; 3 Department of Molecular Biology, Princeton University, Princeton, New Jersey, United States of America; 4 Department of Genome Sciences, University of Washington, Seattle, Washington, United States of America; University of Chicago, United States of America

## Abstract

Maintaining balanced growth in a changing environment is a fundamental systems-level challenge for cellular physiology, particularly in microorganisms. While the complete set of regulatory and functional pathways supporting growth and cellular proliferation are not yet known, portions of them are well understood. In particular, cellular proliferation is governed by mechanisms that are highly conserved from unicellular to multicellular organisms, and the disruption of these processes in metazoans is a major factor in the development of cancer. In this paper, we develop statistical methodology to identify quantitative aspects of the regulatory mechanisms underlying cellular proliferation in *Saccharomyces cerevisiae*. We find that the expression levels of a small set of genes can be exploited to predict the instantaneous growth rate of any cellular culture with high accuracy. The predictions obtained in this fashion are robust to changing biological conditions, experimental methods, and technological platforms. The proposed model is also effective in predicting growth rates for the related yeast *Saccharomyces bayanus* and the highly diverged yeast *Schizosaccharomyces pombe*, suggesting that the underlying regulatory signature is conserved across a wide range of unicellular evolution. We investigate the biological significance of the gene expression signature that the predictions are based upon from multiple perspectives: by perturbing the regulatory network through the Ras/PKA pathway, observing strong upregulation of growth rate even in the absence of appropriate nutrients, and discovering putative transcription factor binding sites, observing enrichment in growth-correlated genes. More broadly, the proposed methodology enables biological insights about growth at an instantaneous time scale, inaccessible by direct experimental methods. Data and tools enabling others to apply our methods are available at http://function.princeton.edu/growthrate.

## Author Summary

A major challenge for living organisms is the regulation of cellular growth in a fluctuating environment. Sudden changes in nutrient availability or the presence of stress factors typically require rapid adjustments of cellular growth. The misregulation of growth control in higher organisms is a major factor in the development of cancer. A statistical characterization of cellular growth based on gene expression levels provides a quantitative perspective to understand the regulatory network that controls growth. We develop a model of cellular growth in the yeast *Saccharomyces cerevisiae*, grounded in the expression levels of a small set of genes. The model is able to predict the growth rate of new cellular cultures from expression data and is robust to changing biological conditions, experimental methods, and technological platforms. The predictions are informative about changes in growth at very short time scales, which direct experimental methods cannot generally access. The model also predicts growth rates in *Saccharomyces bayanus* and in *Schizosaccharomyces pombe*, a yeast diverged by approximately a billion years of evolution. Our findings suggest that the model describes fundamental characteristics of the unicellular eukaryotic growth regulatory program. A case study explores the role of nutrient sensing in the yeast growth regulatory system.

## Introduction

Proper regulation of growth rate is a key systems-level challenge for all cells, particularly microorganisms facing a fast-changing and often hostile environment. Cell growth, defined as an increase in cellular biomass due to biosynthetic processes, is one of the primary functions that must be coordinated with the environment in order for cells to maintain viability and reproduce. The determination of how cells integrate information from the external environment with information from their internal state to mount an appropriate response—growing in the presence of nutrients, arresting growth when stressed, and resuming afterwards— is of central importance to our understanding of basic biology. From a genomic perspective, growth also raises the issue of disentangling correlated systems-level behaviors. When the expression levels of thousands of genes change due to a growth-related stimulus, which underlying regulatory parameters are responsible?

In this paper, we identify quantitative aspects of the transcriptional regulatory mechanisms underlying cellular growth in *Saccharomyces cerevisiae*, and we develop a model to predict instantaneous growth rates of cellular cultures based on gene expression data. The model enables the estimation of growth rates under any conditions for which expression data is available, even on a very short time scale, where standard experimental techniques cannot measure cellular growth directly [1]. For example, a culture undergoing continuous growth in a chemostat [2] can be perturbed from steady state by means of a brief heat pulse, but the departure from and the return to steady state growth is too brief to capture with optical density measurements. Our model allows such a decrease (and subsequent resumption) of growth rate to be quantified under a variety of conditions: batch or chemostat cultures, different microarray platforms, and under any environmental stimulus for which gene expression can be assayed. Surprisingly, this model also successfully predicts growth rates from *Saccharomyces bayanus* and *Schizosaccharomyces pombe* expression data, the latter of which is evolutionarily diverged from *S. cerevisiae* by an estimated billion years [3].

Our findings suggest that the proposed statistical model of cellular growth provides a broadly applicable biological characterization of the transcriptional regulatory network underlying growth rate control. We have previously observed that the expression of ∼25% of the yeast genome responds to changes in growth rate [4]. The response is functionally cohesive, with genes up-regulated with increasing growth enriched for translational and ribosomal functions, and with down-regulated genes enriched for oxidative metabolism and the peroxisome. This functional portrait provides a rich environment in which to study transcriptional regulation of growth; for example, production of new proteins at the ribosome is vital to cellular proliferation, and yeast devotes some ∼60% of its transcriptional throughput to ribosomal RNA [5]. Similarly, growth rate regulation is highly interconnected with a variety of other cellular processes (e.g. the environmental stress response [6], metabolic cycling [7], and the cell cycle [8]), and we discuss potential causative regulatory signals from the Ras/PKA pathway [9] and growth-related transcription factors.

Our recent analysis of gene expression measurements from a collection of *S. cerevisiae* chemostat cultures across several nutrient limitations and growth regimes [4] offered intriguing evidence for a notion of instantaneous growth rate. In this paper, we develop a model to characterize such a notion quantitatively, in a statistically principled fashion. We further assess the robustness of the proposed characterization by presenting new computational evidence on six additional published data sets and on four newly collected data sets. More in detail, we demonstrate that the model can accurately predict relative growth rates under a variety of conditions and is robust to the conditions of the originating culture, the technological platform used to assay gene expression, and evolutionary conservation to other organisms (*S. bayanus* and *S. pombe*). The model allows us to predict growth rates for published genome-wide collections of expression data (e.g. the stress response [6] or gene deletions [10]) and for four new data collections we have generated for this work (Tables S1, S2, S3, S4), providing biological insight into the growth rate response at very short time scales—minutes, rather than the hours necessary to experimentally assay doubling times. This biological validation of the predictions is accompanied by an out-of-sample validation and outlier analysis to assess the statistical accuracy of the model. We have made an implementation of this model available to the public at http://function.princeton.edu/growthrate.

Additional analyses offer biological insights that support and further substantiate the empirically observed robustness of the predictions based on the newly characterized growth-rate genes. Our insights rely, in part, on the quantitative identification of binding motifs of known (and uncharacterized) transcription factors associated with the genes responding to growth. Moreover, our model enables a quantitative characterization of growth profiles underlying puzzling experimental evidence that provides a first convincing explanation of observed cell death in response to a perturbation in the Ras/cAMP/PKA pathway. More in detail, we apply our model to study two important aspects of cell growth regulation: nutrient sensing and the cell cycle. Artificial activation of the Ras/cAMP/PKA pathway has been previously observed to recapitulate approximately 85% of the expression response associated with increased growth in the presence of glucose [11]; here, we show that the cell's regulatory state during this activation is indicative of an up-regulated growth response, even in the absence of appropriate nutrient availability. This conflict between internal regulatory state and the external environment leads to rapid cell death. In contrast, analysis of growth rate regulation during metabolic cycling [12] and synchronous cell cycles [8],[13] indicates that growth rate regulation is not specific to cell cycle phases, but it is strongly limited to the oxidative phase of the metabolic cycle. These observations, coupled with an analysis of putative transcription factors mediating the growth response, establish a substantial foundation on which to base further experimental work on the systems-level control of cellular growth rate.

### Background: Measuring Growth

**Figure 1:**
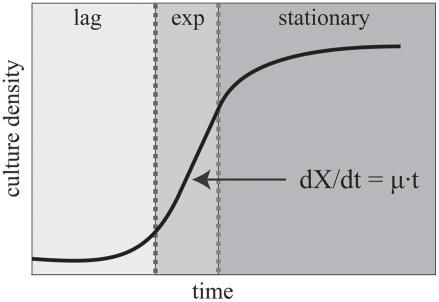
Growth phases of a typical cellular culture.

Cellular growth is typically quantified in one of two experimental environments: batch culture or the chemostat. In a batch culture, cells are provided with a saturating amount of nutrient [1]. Growth is quantified by measuring the optical density (OD) of the culture over time, X. Figure 1 illustrates three typical phases of an OD growth curve: a slow initial phase (lag), a fast exponential growth phase (exp), and a slow saturation phase (stationary). Solving the appropriate differential equation leads to an exponential model of cellular growth, X = e ^μ·t^. In practice, the OD of a culture is sampled at discrete points over time, and the growth rate parameter μ (in units of inverse hours h^−1^) is estimated from an exponential fit to the OD measurements.

**Figure 2:**
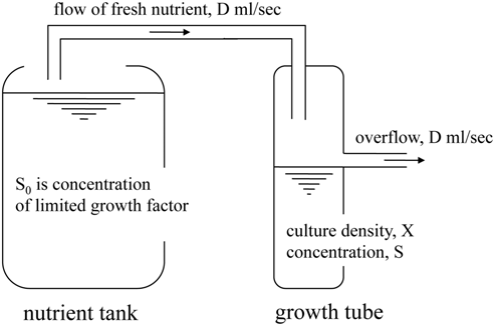
Schematic of a chemostat. In the chemostat, cells are grown in liquid media [14]. A tank contains a large supply of nutrient containing high concentrations of all growth factors, but a limited concentration (S_0_) of the controlling growth factor. The nutrient flows continuously into a growth tube of limited capacity, where the culture grows. The dynamic behavior of the density of the culture (X) and of the concentration of the controlling nutrient (S) in the growth tube is summarized with a system of Michaelis-Menten differential equations. The desired growth rate is attained by manually limiting the concentration of the controlling growth factor in the nutrient provided to the cells.

In the chemostat, a specific growth rate is maintained by limiting the concentration of a controlling nutrient provided to the cells [14]. Figure 2 illustrates the principle behind the chemostat. A limited concentration (S_0_ in the tank) of the controlling growth factor is provided in media flowing continuously into a growth tube of limited capacity. Changes in density of the culture, X, and in concentration of the controlling nutrient (S), in the growth tube, are driven by Michaelis-Menten dynamics [15]. In this regime, the growth rate is a function of the concentration of the controlling nutrient, μ = μ(S). In particular, dX/dt = [μ(S)−D] X; at steady state, the density of the culture no longer changes, dX/dt = 0, and the concentration of the controlling growth factor also stabilizes, dS/dt = 0. The growth rate then equals the flow rate set by the experimenter, μ(S*) = D.

In a batch culture, the growth rate is generally not controlled; it is determined by a complex interaction of environmental and genotypic states, and it is maximal during the exponential phase of growth (μ_max_). Under these conditions, the growth rate of the culture (the first derivative of the curve in Figure 1) changes with time. In a chemostat, the growth rate is controlled by setting the nutrient flow rate D below an organism's maximum possible growth rate μ_max_ as estimated from batch culture. In either experimental environment, the growth rate μ is directly related to the doubling time, T_d_ = ln(2)/μ.

Our model is built on a collection of gene expression data from chemostats at known growth rates, and it allows us to quantify a notion of instantaneous growth rate in chemostat and batch cultures, even in cultures in which the growth rate is changing rapidly over time.

## Materials and Methods

We fit a linear model to a collection of expression data drawn from *S. cerevisiae* chemostat cultures over several growth rates and nutrient limitations. Estimates of the parameters characterize each gene's response to changes in growth rate, and provide insight into the transcription factors and regulatory network responsible for yeast growth homeostasis. By applying this model to new expression data sets, we are able to predict instantaneous growth rates for any yeast culture. The model is robust to the biological and technical conditions of the originating gene expression data and enables the prediction of growth rates at instantaneous time scales inaccessible to standard experimental methods (e.g. optical density). We have also successfully applied the model to the related organisms *S. bayanus* and *S. pombe*. Data and tools relating to this model are made available at http://function.princeton.edu/growthrate.

### Experimental Design and Data

Our model is based on a collection of gene expression measurements from steady state (chemostat) cultures limited across several nutrients and growth regimes. Briefly, 36 CEN.PK derived *S. cerevisiae* chemostat cultures were grown under six nutrient limitations: Glucose (G), Nitrogen (N), Phosphate (P), Sulfur (S), Leucine (L), and Uracil (U). Six growth rates were used for each nutrient, ranging by steps of 0.05 h^−1^ from 0.05 h^−1^ to 0.3 h^−1^. Agilent Yeast V2 microarrays were used to measure gene expression in the resulting 36 chemostats; for details, see [4]. This experimental design provides the opportunity to discover gene expression patterns correlated with growth rate, independently of nutrient-specific responses.

**Figure 3:**
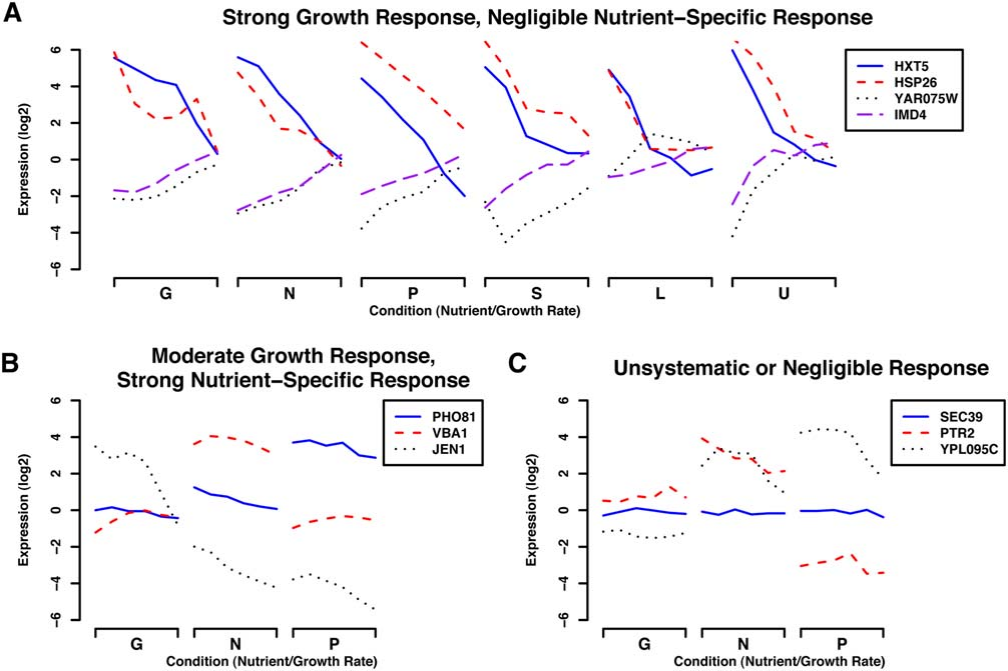
Representative genes responding to growth rate, specific nutrients, or unsystematically in our chemostat-derived training data. Our statistical model of growth rate regulation is based on expression data collected from 36 chemostats at six growth rates (0.05 hr^−1^ through 0.3 hr^−1^) under six nutrient limitations (Glucose, Nitrogen, Phosphate, Sulfur, Leucine, and Uracil) as described in [4]. By employing the genes responding strongly, consistently, and only to changes in growth rate (and not specific nutrients) as growth-specific genes, we can apply our model to predict relative growth rates in new expression data. Gene expression in our original 36 conditions fell into three main categories as shown here. (A) Genes strongly up- or down-regulated in response to changes in growth rate, independent of limiting nutrient. The most statistically significant members of this set became our growth-specific calibration genes for application of the linear model to other expression data. (B) A subset of conditions highlighting genes with expression levels showing some correlation with growth rate, but with a strong nutrient-specific component. This represents a sizeable portion of the genome (∼25%), with positively growth-correlated genes enriched mainly for ribosomal function and negatively correlated genes enriched for oxidative metabolism. (C) A subset of conditions highlighting genes showing a non-systematic or negligible change in gene expression. Unresponsive genes were enriched for a variety of cellular processes not expected to show a strong relationship with growth, e.g. transcription, DNA metabolism and packaging, secretion, and many others.

Figure 3 highlights the sources of variability in the gene expression profiles that the experimental design aims at capturing. The resulting data contain a number of characteristic gene expression patterns, including genes with strong growth-specific transcriptional regulation and negligible nutrient-specific response (Figure 3A). Other genes include a growth-specific expression component but are also strongly up- or down-regulated under specific nutrient limitations (Figure 3B). Finally, Figure 3C displays expression profiles that show unsystematic or negligible responses under these conditions. The linear model described below summarizes the variability in the expression profiles of individual genes specifically due to changes in growth rate, which leads to a characterization of *growth-specific calibration genes* such as those shown in Figure 3A. This growth-specific signature enables predictions of the instantaneous growth rate of any cellular culture based on the relative expression values these growth-specific genes.

Table 1 summarizes the collections of expression data analyzed in this study. Six collections were previously published by others, one was published in our previous work [4], and four are new to this study: 1. chemostats limited for different nitrogen sources, 2. heat pulses inducing a temporary departure from steady state growth, 3. artificial activation of the Ras/PKA pathway, and 4. *S. bayanus* diauxic shift and heat shock time courses. All gene expression collections were pre-processed as in [16]. The gene expression values for all growth-specific genes are provided in Tables S1, S2, S3, S4, respectively.

**Table 1:** Overview of expression data analyzed in this study.

Experimental Conditions	Method	Platform	Organism	Publication/Experimenter
Nutrient-limited growth	Chemostat	Agilent	*S. cerevisiae*	[4]
Cell cycle synchronization	Batch	Spotted	*S. cerevisiae*	[13]
Cell cycle synchronization	Batch	Spotted	*S. cerevisiae*	[8]
Metabolic cycling	Batch/Chem.	Affymetrix	*S. cerevisiae*	[12]
Environmental stress	Batch	Spotted	*S. cerevisiae*	[6]
Gene deletion mutants	Batch	Spotted	*S. cerevisiae*	[10]
Heat pulses	Chemostat	Agilent	*S. cerevisiae*	C. Lu, Table S1
Nitrogen-limited growth	Chemostat	Agilent	*S. cerevisiae*	D. Gresham, Table S2
RAS/PKA activation	Batch	Agilent	*S. cerevisiae*	J. R. Broach, Table S3
Diauxic shift, heat shock	Batch	Spotted	*S. bayanus*	A. A. Caudy, M. J. Dunham, Table S4
Hydroxyurea response	Batch	Spotted	*S. pombe*	[29]

Of the 11 gene expression data sets for which we predict and discuss growth rates, four are previously unpublished; excerpts of this data relevant to the growth rate analysis are provided in Tables S1, S2, S3, S4. These data span various experimental conditions, dual- and single-channel expression array platforms, batch and steady-state growth regimes, and three species of yeast. Under these varied conditions, our growth model predicts instantaneous growth rates and provides insight into regulatory mechanisms for growth homeostasis.

### Linear Models and Identification of Growth-Specific Signature

We sought to identify a small set of genes providing a quantitative summary of cellular growth rate regulation. Genome-wide expression measurements underlying the 36 chemostat cultures provided us with the opportunity to determine which genes were responding linearly to changes in growth rate, and not to differences in nutrient limitation. To identify such gens in a statistically principled fashion, we performed four steps, beginning by using maximum likelihood to fit a linear model of each gene *g*'s expression under all training conditions (**Y** *_g_*) based on the conditions' known growth rates (**X** *_c_*):
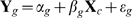
(1)


This step yields two learned parameters per gene, a baseline expression level *α_g_* and a growth rate response *β_g_*. The model is fit to minimize the residual error **ε** *_g_*, which can capture either non-growth-related biological variability or technical noise. We fit this model for the yeast genome using the expression levels from our 36 chemostat conditions, recording each gene's *α_g_* and *β_g_* parameters and its goodness of fit (total explained variability) R^2^ *_g_*.

We next used the bootstrap (i.e. a randomized re-sampling technique) to assess the expected background distributions of these parameters in the absence of a growth-related biological signal (i.e. the null distributions). We constructed 100,000 randomized expression vectors of length 36 by sampling each component (with replacement) from the collection of gene expression values in the corresponding condition, i.e., the same combination of growth rate and nutrient limitation. For example, the first value randomly chosen for such a vector could be drawn from any gene or nutrient limitation in our chemostat data at a flow rate of 0.05 h^−1^, the second from any flow rate of 0.1 h^−1^, and so forth. Note that by re-sampling the expression values of putative genes column-by-column, we do not wash away the average transcriptional response that is expected to be associated with nutrient-growth rate pairs. In this sense, the null distribution we derive carries information about how genes respond to growth across nutrient limitations, on average. As a consequence, the statistical significance of the differential response we compute is biologically justified. In other words, this sampling scheme maintains average nutrient specific and growth rate specific information, and leads to an estimate of the null distribution in the absence of gene-specific growth related and nutrient related gene expression. This process yields null distributions for parameters *α_g_*, *β_g_*, and the goodness of fit R^2^ *_g_*.

Third, from these null distributions, we assign false discovery rate corrected p-values [17] to each gene's *α_g_*, *β_g_*, and R^2^ *_g_* values. Finally, a gene was deemed to have a significant expression response to changes in growth rate if it fit this model well (R^2^ *_g_* p<0.05) and was up- or down-regulated significantly with growth (*β_g_* p<0.05); this information is available in [4]. We further characterized a specific set of *growth-specific calibration genes* responding only and significantly to changes in growth rate (*β_g_* p<10^−5^ and R^2^ *_g_* p<10^−5^) that we used to infer instantaneous growth rates in new expression data (Table S5 and Dataset S1).

### Model-Based Prediction of Instantaneous Growth Rates from Expression Data

The set of growth-specific genes identified with the four-step procedure above represents a quantitative signature of a cellular culture's transcriptional regulation of growth rate, i.e. the speed at which cells are proliferating. By examining these genes' expression levels in a new collection, we can predict the instantaneous growth rate of the cellular culture the expression measurements correspond to. This notion of instantaneous growth rate is comparable to the derivative of an optical density growth curve, but it can be inferred robustly by our model on any time scale, e.g. minutes, from expression data, without the need to measure one or more full doubling times of a culture.

Given expression data for a new experimental condition, we use an iterative maximum likelihood approach to infer its growth rate using the parameters captured by our linear model. Formally, consider a vector of expression measurements for *n* growth-specifc genes, **Z**_1:*n*_. As described above, the expression of these growth-specific genes varies primarily in response to changes in a condition's growth rate, which we model as the mean *μ* of a Gaussian with variance *σ* ^2^. Using our previously calculated maximum likelihood estimates of the calibration gene parameters **α**_1:*n*_ and **β**_1:*n*_, the expected value of a gene's expression is thus:
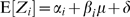
(2)


Here, *δ* is a condition-specific parameter that captures the condition's baseline gene expression, i.e. an average offset between a new experimental condition and our training expression data. In dual-channel data, this parameter may capture differences between a new condition's reference channel and our training data's reference channel; for a single-channel array, *δ* may capture the absolute difference between the platform baseline and our training data. In any event, the expected variability of a new measurement is:
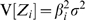
(3)


The likelihood of the expression measurements **Z**_1:*n*_ is thus a product of Gaussians:
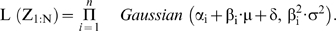
(4)


From this, we derive the maximum likelihood estimate of the condition's growth rate *μ*_ML_:
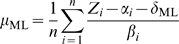
(5)


Similarly, the maximum likelihood estimate of the condition's baseline *δ*_ML_ is given by:
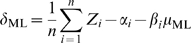
(6)


Note that the estimate of *δ*_ML_ depends on the estimate of *μ*_ML_, and vice versa. To calculate these estimates, we initialize *μ*_ML_^(0)^ assuming *δ*_ML_^(0)^ = 0 and iterate subsequent computations of *μ*_ML_^(*t*+1)^ and *δ*_ML_^(*t*+1)^ to convergence. In practice, individual growth-specific genes with residuals outside the inner fences of all growth-specific gene residuals (more than 1.5 inter-quartile ranges from the lower or upper quartiles, [18]) are noted as outliers and do not participate in that condition's growth rate inference procedure. This allows outlier genes responding to non-growth related stimuli (which are, in general, infrequent, e.g. six in one of our most variable conditions as discussed below) to be noted for further investigation, while also decreasing the cross-validated error of predicted growth rates.

### Extending Predictions to Additional Organisms

In principle, this model of growth rate can be extended to study and predict instantaneous growth in any organism for which appropriate homology to growth-specific genes exists. To analyze growth rates in expression data from *S. bayanus* and *S. pombe*, the *S. cerevisiae* calibration genes were mapped to known orthologs. This mapping was performed using the unambiguous pairings from [19] for *S. bayanus* and the curated orthologous groups from [20] for *S. pombe*. This resulted in 51 growth-specific genes for *S. bayanus* and 74 for *S. pombe*, the increase being due to one-to-many mappings; see Table S5.

### Online Tool Availability

The parameter estimates driving our predictions and tools allowing users to predict growth rates in new data sets are available at http://function.princeton.edu/growthrate. Specifically, users can upload *S. cerevisiae* expression data (single- or dual-channel in standard PCL format) to receive estimates of relative growth rate for each condition. If a reference with known growth rate is provided, absolute rate estimates will be generated. This growth rate prediction tool has been implemented in R and is also available for offline use, allowing further customization (such as application to additional organisms).

## Results

We apply our linear model of growth rate regulation to predict instantaneous growth rates for a variety of expression data. This includes new chemostat cultures used to assess prediction quality, publicly available stress response and gene deletion microarrays from batch cultures, growth differences between metabolic cycling and the cell cycle, several different microarray platforms, and an out-of-sample validation to quantify model accuracy. We also observe good predictive performance for growth rates in *S. bayanus* and *S. pombe* data sets, the latter despite up to a billion years of evolutionary divergence from our *S. cerevisiae* training data. This suggests that the growth-related transcriptional regulation captured by our model is a key feature of unicellular homeostasis, a feature we explore by examining nutrient sensing inputs through the Ras/PKA pathway and potential growth rate transcription factors and binding sites.

### Relative Growth Rate Prediction in Novel Experimental Settings

**Figure 4:**
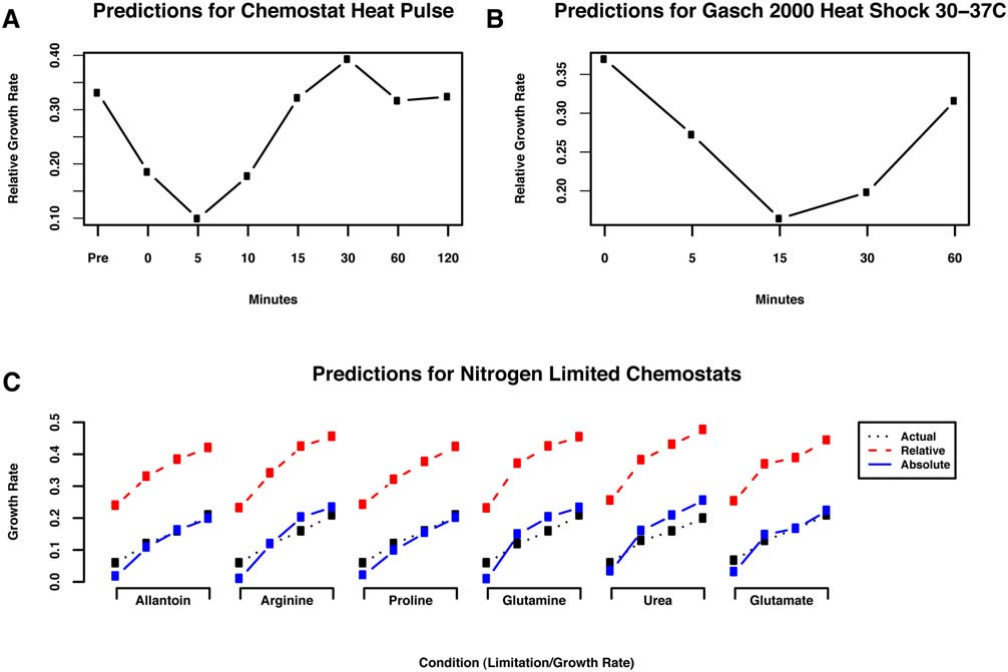
Predicted growth rates for *S. cerevisiae* gene expression datasets. Our model of the growth rate transcriptional response can be used to predict the growth rate of a cellular culture from gene expression data, robust to the originating biological conditions, growth regime, and experimental platform. Here, we apply the model to three selected data sets to infer relative and absolute growth rates. (A) A brief (<30 s) heat pulse was administered to a steady state chemostat culture immediately before time zero, and gene expression was assayed with an expression time course (see Figure S1 and Table S1). The relative growth rates inferred from this data show an abrupt departure from steady state growth, followed by a return to steady state (including a brief regulatory overshoot). Our predictions monitor these changes in growth rate at an instantaneous time scale (<5 m) inaccessible by standard experimental assays for growth rate. (B) Predicted growth rates for a portion of the environmental stress response data [6], assaying the response to a 30–37°C heat shock. Our model captures the cessation and resumption of growth induced by the stress, even for a batch culture in which the growth rate is not fixed a priori. (C) A collection of 24 chemostats were run at four growth rates (0.05 hr^−1^ through 0.2 hr^−1^) and limited on six different nitrogen sources. Using only expression data from each condition, our model predicts accurate relative growth rates. However, when provided with the known growth rate for a single condition, the model is additionally able to infer absolute growth rates for all other data sets sharing that condition's mRNA reference channel. Note that the actual growth rate is measured empirically and thus deviates slightly from an ideal straight line due to technical variation in the growth equipment.

Our model of the growth rate transcriptional response can be used to predict relative instantaneous growth rates from any *S. cerevisiae* gene expression data. For example, Figure 4A shows our predicted growth rates for a gene expression time course sampled from a steady state culture exposed to a brief (<30 s) heat pulse (Table S1). The predictions clearly show a departure from steady state within five minutes of the heat pulse, followed by recovery within 15 minutes. Similar predictions over a range of chemostat flow rates (Figure S1) reveal that this cellular behavior is consistent, although there is some variation in the degree of growth cessation during stress, in agreement with tolerance and sensitization models of the yeast stress response [21]. Notably, standard experimental assays for growth rate (e.g. optical density) would be incapable of monitoring such a response, while our model is able to observe these growth changes on an instantaneous time scale.

A similar application of our model to predict relative growth rates for the stress response conditions of [6] is presented in Figure 4B (see Figure S2 for complete results). These data represent yeast batch cultures assayed using a variety of different reference mRNA samples on a custom spotted microarray platform, none of which differences from our training data impair the growth rate estimation process. While there are no direct measurements of growth rate in these non-steady-state conditions, our predictions are consistent with known yeast biology and agree with expected growth behavior. Most shock time courses, including all heat shocks, peroxide, diamide, and hyper-osmotic stress, provoke an initial sharp decrease in growth rate followed by a return to initial or near-initial rate; shorter shocks, such as DTT, menadione, and peroxide responses, capture only the rate decrease. Batch growth proceeds at a fairly constant rate until nutrients become depleted, at which point the rate decreases sharply; this pattern is also seen in intentional nitrogen depletion. Growth rates across varying temperatures peak as expected at 25 C [1], falling off at lower and higher temperatures. Finally, response to varying carbon sources is also as expected [22], with ethanol inducing the slowest growth and fructose, sucrose, and glucose allowing the most rapid. Our model's inference of growth rate from gene expression data alone allows both post hoc growth analysis (e.g. years after the original experiment) and an estimation of growth rates for cultures where direct growth measurements would be unfeasible, difficult, or time consuming.

When applied to expression data from yeast mutant strains, in which one or more genes have been deleted, predicted growth rates can be used to quantify single mutant fitness. We used our model to analyze the knockout collection assayed in [10]; predictions on the complete data set are available in Table S6. Direct fitness measurements for 199 of the ∼300 mutants assayed via microarrays is available as supporting information [10]. Our predictions for these 199 growth rates correlate very strongly with the direct fitness measurements (ρ = 0.473, p<10^−11^) and are derived solely from expression data. In contrast, methods for experimentally estimating single mutant fitness from high-throughput growth curves showed substantially less agreement (ρ = 0.321, p<10^−6^ [23]; ρ = 0.108, p>0.2 [24]) with the original publication's direct fitness measurements. These results represent a compelling argument as to the relevance of our growth rate model for fitness estimation.

### Absolute Growth Rate Prediction with One Shared Reference

With a small amount of additional information (i.e., a scalar) the relative growth rates inferred by our model can be made absolute, in units of chemostat flow rate (hr^−1^). Our model's predicted rates for a collection of arrays are relative estimates, to one another. This is due to the unknown quantitative effects of the reference mRNA in our dual-channel training data; it is impossible to know a priori the relationship between this reference channel and the relative (for dual-channel) or absolute (for single-channel) expression levels in new microarray data. However, if the absolute growth rate is known for some array in a given collection, our model can make absolute rate predictions for other two-color arrays in the collection, given that they all share the same reference channel.

Figure 4C shows actual growth rates (dotted, black lines) for a collection of chemostats at various flow rates limited on one of several different nitrogen sources (Table S2) along with estimates of the relative instantaneous growth rates (red, dashed lines) and of the absolute instantaneous growth rates (solid, blue lines). Absolute growth rates are estimated by recording the growth rate in the Proline limited chemostat at μ = 0.35 hr^−1^, and shifting all the estimates accordingly, since the dual-channel microarrays in this study all share the same transcriptional readout in the reference channel. We sought to evaluate the goodness of the predictions in Figure 4C by computing the statistical significance of their correlation with the actual growth rates. To this end, we computed the correlation between the true growth rates and the predicted instantaneous growth rates. The correlation is the same for both absolute and relative predicted rates, as they differ by a constant, and equals ρ = 0.956 (p-value≈0). This computation provides statistical support to the goodness of the predictions produced with the proposed model. More in general, on normalized dual-channel microarrays, the doubling of any gene's mRNA level in these conditions results in the same increase in its expression readout. Thus one unit of predicted relative rates to corresponds to one unit of absolute chemostat flow rate. However, since the reference channel differs from that of the arrays used to train the model, all rate predictions are typically off by a corresponding constant factor. By normalizing to any one of the *N* arrays' known growth rates, this shift can be automatically corrected for the *N-1* other arrays, employing the same reference channel.

### Accuracy of the Predictions and Outlier Detection

**Figure 5:**
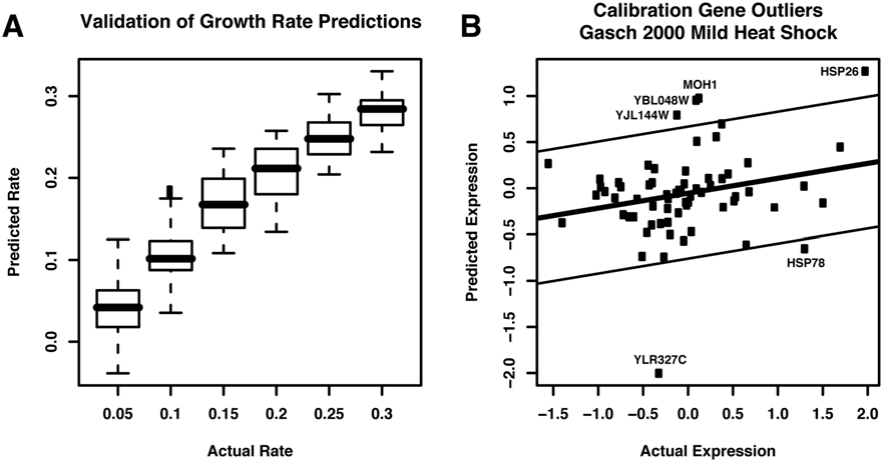
Assessment of accuracy and outlier detection during growth rate inference. (A) We performed an out-of-sample cross-validation of our model by randomly sub-sampling 24 of the 36 training expression arrays 1,000 times. We refit our linear model in each random sample, calculated bootstrapped null distributions for all gene parameters, and found sets of the most significant growth-specific genes. These were then used to infer growth rates for the 12 held-out conditions, providing an estimate of the accuracy of the model's growth rate predictions. (B) When predicting the growth rate of a new collection of expression data, our model excludes any calibration gene with an expression level outside the inner fence (1.5 times the inter-quartile range below or above the first or third quartiles). This improves predicted growth rate accuracy while also calling out genes potentially responding to specific non-growth stimuli under some biological condition. For example, in the [6] mild heat shock time course, two of the six outliers are known heat shock genes (HSP26 and HSP78). The other four (YLR327C, MOH1, YBL048W, and TMA10) are uncharacterized genes, suggesting potential roles in the response to heat shock.

We assessed the quality of our growth rate predictions using 1,000 out-of-sample experiments, according to a hybrid bootstrap/cross-validation setup, using the data from [4]. Results are shown in Figure 5A. In each experiment, we randomly withheld 12 of the 36 conditions for testing, fit our linear model on the remaining 24, derived bootstrapped null distributions using only these data, and determined growth-specific gene sets to use for growth rate inference on the held-out conditions. This experimental setup leads to absolute growth rate predictions directly, as all the dual-channel microarrays share the same transcriptional readout in the reference channel. This out-of-sample validation allowed us to assess the accuracy and variability of our predictions on conditions with known growth rates not included in the model building procedure. In addition to the performance indicated by Figure 5A, the out-of-sample experiments demonstrated robustness of p-value cutoffs and number of growth-specific genes; these ranged in number from ∼50 to ∼110 across the randomized validations (of a total ∼5,500 possible genes), and changes of this magnitude in the final calibration gene set had little impact on predicted growth rates. We further quantified a notion of reliability for each of the 72 growth-specific genes. Specifically, we computed the percentage, *P*, of bootstrap experiments in which each individual gene was selected as a member of the growth-specific gene set. The percentages provide an expectation about whether each individual gene should be considered reliable in a new study. We found that 69 genes were selected in more than half of the experiments, *P*>*0.5*. Full results are reported in Table 2.

**Table 2:** Reliability study for the 72 growth-specific genes.

Gene	Percentage	Gene	Percentage	Gene	Percentage	Gene	Percentage
UTR2	1	NOP1	0.78	HSP26	0.98	HSP30	0.83
AMS1	0.96	OLI1	1	DCS2	0.97	GND2	0.87
CTP1	0.64	SNO4	0.97	GSC2	0.84	RPL24A	0.62
YOL014W	1	RPL7A	0.97	FUR1	0.57	OM45	0.77
HXT5	1	HSP78	0.67	MOH1	1	BTN2	0.63
YPT53	0.99	RPL18A	0.89	RPL20A	0.67	POT1	0.74
YJR008W	0.61	NCA3	1	YHR138C	0.6	RPL31B	0.69
HSP32	0.96	HSP42	1	RPP2A	0.89	NDE2	0.75
GPG1	1	MSC1	1	YLR312C	1	UGX2	0.63
YBL048W	1	YDR379C-A	0.57	UIP4	0.96	YDR070C	0.8
DDR2	0.99	PAI3	0.95	YGR043C	0.97	PHM7	0.76
YJL161W	0.94	TFS1	0.79	YLL067C	0.63	CTT1	0.7
RPL23A	0.65	RPP1A	0.89	YMR196W	0.93	HSP12	0.62
YOR338W	0.62	ROM1	0.56	SPG1	0.84	GPH1	0.63
RPL18B	0.82	RPP1B	0.81	PET10	0.8	SOL4	0.68
SSE2	0.97	SNU13	0.64	RPS28A	0.79	YIR016W	0.37
HSP104	0.97	YBR116C	1	YLR327C	0.86	GRE1	0.44
AAC3	0.69	IMD4	0.96	YTP1	0.58	GDH2	0.28

Reliability for an individual gene was quantified by computing the percentage of 1,000 bootstrap experiments where the gene was selected in the set of growth-specific genes. Results suggest that 69 genes are expected to be reliable in a new study, *P*>0.5.

In the process of estimating growth rates and determining this confidence score, growth-specific genes with outlying expression values are also detected. While most conditions induce few outlying growth-specific genes, when they occur, they are *not* indicative of the quality of growth rate predictions. We have found that neither the number of outliers nor their variability correlates with prediction error (data not shown), but they call out genes that may be responding to non-growth stimuli under specific biological conditions. Excluding outliers from the growth rate estimation process improves the accuracy of the predictions, and these outliers can in turn be biologically informative: an outlying growth-specific gene is likely responding specifically to a stimulus other than change in growth rate. For example, some of the only outliers in the mild heat shock time course from [6] occur towards the end of a shift from 29 C to 33 C (Figure 5B). These include *HSP26* and *HSP78*, both known heat shock chaperones [25],[26]. Three genes of unknown function (*YLR327C*, *MOH1* and the neighboring dubious ORF *YBL048W*, and *TMA10*) are also outliers in this condition, which is evidence that these genes may have specific expression responses (and thus biological functions) during heat shock. *HSP26* and *YLR327C* are frequent outliers in stress-related conditions, perhaps suggesting a more general stress response function.

### Predicting Growth Rates in *S. bayanus* and *S. pombe*

**Figure 6:**
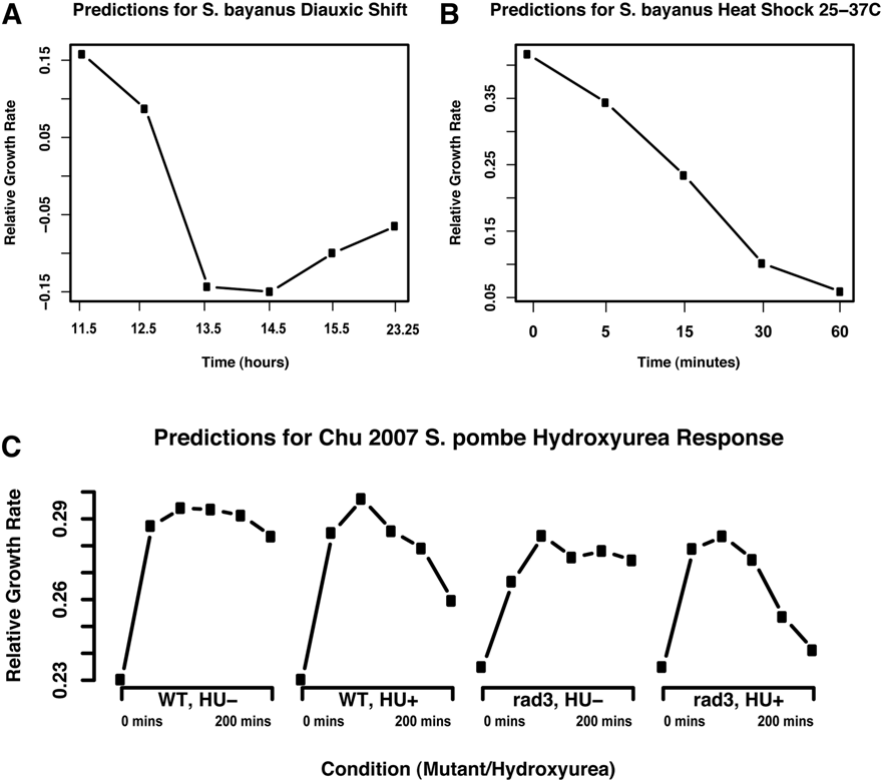
Predicted growth rates for *S. bayanus* and *S. pombe* expression datasets. By examining genes orthologous to our ∼70 *S. cerevisiae* growth-specific calibration genes, we successfully applied our model to predict growth rates in *S. bayanus* (∼50 orthologous growth-specific genes, ∼20 M years diverged) and *S. pombe* (∼75 growth-specific genes due to one-to-many mappings, ∼1B years diverged). (A) Predicted growth rates for *S. bayanus* undergoing the diauxic shift from fermentative to respiratory growth (Table S3). As observed for the *S. cerevisiae* diauxic shift in [4], growth pauses as glucose is exhausted and resumes as the yeast begins consuming ethanol. (B) Predicted growth rates for S. bayanus exposed to a 25–37 C heat shock (Table S3). In contrast to Figure 4B, in which *S. cerevisiae* is observed to recover from a 37 C heat shock, the less-thermotolerant S. bayanus [28] is predicted to halt growth at high temperatures. (C) Predicted growth rates for S. pombe wild-type and rad3Δ time courses, grown normally and exposed to hydroxyurea (HU, an inhibitor of DNA synthesis and thus growth) [29]. Despite the wide evolutionary divergence between *S. pombe* and our *S. cerevisiae* training data, predicted growth rates are in substantial agreement with expected biology. Each time course begins with low growth in a synchronized culture. When the synchronization block is released, cells begin growing, wild-type more efficiently than the rad3Δ mutant. Exposure to HU decreases growth over time, and this effect is exacerbated by RAD3 deletion. While the *S. cerevisiae* RAD3 ortholog MEC1 is essential, knockouts of the MEC1 pathway members SOD1 and LYS7 have been previously observed to induce HU sensitivity [30].

While our growth rate model is based on a transcriptional growth signature in *S. cerevisiae*, the model can be applied to any organism with sufficiently orthologous transcriptional activity. This is likely to be the case within the *sensu stricto* yeasts, separated by ∼25 million years of evolution [27]. By finding the ∼50 *S. bayanus* genes orthologous to our ∼70 *S. cerevisiae* growth-specific calibration genes [19], we can apply our model directly to *S. bayanus* expression data (Table S4). Figure 6 demonstrates such a result for two *S. bayanus* time courses assaying the diauxic shift and a response to heat shock. These results have comparable profile to those from *S. cerevisiae* and are similarly biologically compelling. For example, the diauxic shift in *S. bayanus* results in a very similar growth pattern to the known response in *S. cerevisiae*, with a near-cessation of growth during the shift and subsequent rebound. Conversely, *S. bayanus* is less resistant to high temperatures than *S. cerevisiae* [28], and our growth rate inferences show a corresponding failure in its ability to grow following severe heat shock.

We have also extended our model to a significantly further diverged yeast, specifically the yeast *Schizosaccharomyces pombe*, separated from *S. cerevisiae* by an estimated one billion years of evolution [3]. A mapping of our growth-specific calibration genes to *S. pombe* using information from [20] results in ∼75 genes due to one-to-many correspondences, but these provide sufficient calibration information to make high quality predictions (Figure 6C). Calibration gene outliers and expression cohesiveness are not substantially changed relative to *S. cerevisiae* and *S. bayanus*, and the inferred relative rates reflect various biological expectations. All cultures (data from [29]) show an initial increase from low growth rates due to stalled growth during synchronization. An expected decrease in growth rate is predicted during increased exposure to hydroxyurea (HU), and a *rad3*Δ deletion (*S. cerevisiae* ortholog *MEC1*) incurs a mild overall growth impairment as well as exacerbating HU sensitivity. While *MEC1* is essential in *S. cerevisiae*, this sensitivity has previously been noted for deletions *sod1*Δ and *lys7*Δ, both members of the MEC1 pathway [30], which is necessary for the cell cycle checkpoint function.

The extent to which transcriptional regulation is conserved between *S. cerevisiae* and *S. pombe*, which allows us to successfully apply the model despite the evolutionary distance that separates these species, is reflective of cellular growth's central role, particularly in unicellular organisms. While this model would become less meaningful in metazoans, where the growth of individual cells is subjugated to the growth and differentiation of the organism as a whole, certain transcriptional growth behavior is of necessity conserved in single celled organisms [31]. This is particularly true of the ribosome, one of the main contributors to our model's predictive power; rRNA regulation is purely transcriptional, and ribosomal proteins must be expressed stoichiometrically. Since any cellular growth requires translation, observation of ribosomal transcription is a strong indicator of unicellular growth [5]. This is one aspect of the transcriptional growth response made quantitative by our model.

### Insights into Growth Homeostasis

To further investigate the biological basis of growth rate correlated gene expression, we used our model to predict relative growth rates for two interesting cases: the yeast metabolic cycle [12] and the mitotic cell division cycle [8],[13]. The expression data published by Tu et al. was obtained for cells grown at high density in a glucose-limited chemostat. Under this regime, cells within the culture become metabolically synchronized and undergo periodic consumption of oxygen (defined as the oxidative phase of the metabolic cycle) followed by periods of undetectable oxygen consumption (termed the reductive building and reductive charging phases). The cell cycle data sets by Spellman et al and Pramila et al were obtained from experiments in which cells were uniformly arrested in the cell division cycle using a variety of methods and then released to undergo synchronous cell division cycles.

**Figure 7:**
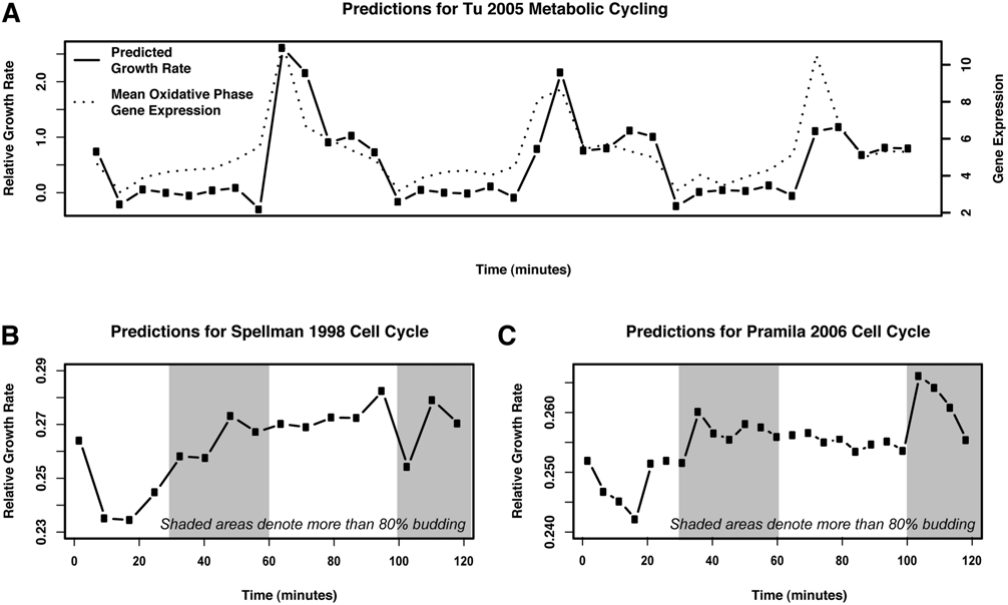
Differences in growth characteristics of a metabolically cycling culture compared to cells synchronously undergoing the cell division cycle. We predict periodic bursts of growth during the oxidative phase of the metabolic cycle as described by [12]. Conversely, we observe essentially no variation in growth in cultures synchronously undergoing the cell division cycle, which has been shown to primarily occupy the reductive phase of the metabolic cycle [32]. (A) In cells undergoing metabolic cycling, growth rates are predicted to peak during the oxidative phase of the cycle, where [12] also observes strong upregulation of translational and ribosomal genes. (B) The predicted growth rate for the [13] alpha-factor synchronized cell cycle is essentially constant, after an initial release from the synchronization block. (C) Predicted rates for the [8] alpha-factor synchronized cell cycle also show an initial resumption of growth after alpha-factor block followed by relatively constant growth rate. Taken together, these observations support the claim that growth rate regulation is not specific to any one cell cycle phase. This also agrees with the fact that rapidly growing (and thus fermenting) *S. cerevisiae* does not partition metabolism into discrete stages, a phenomenon only occurring when reductive metabolism is hindered by nutrient limitation or other stresses.

Growth rate prediction applied to the yeast metabolic cycle data revealed a striking periodicity (Figure 7A). The cyclical pattern of growth rate variation occurs completely in concert with the metabolic cycle as defined by Tu et al. Specifically, the culture's growth rate is predicted to be at minima during the reductive phase of the metabolic cycle, when oxygen consumption is at a minimum, and reach maxima during the peak of the oxidative phases when oxygen consumption is maximal. In contrast, growth rate prediction for the cell cycle (Figure 7B and 7C) show virtually no variation in predicted growth rate during the different stages of cell division.

These data support and extend our previous assertions [4] that the there is a close connection between the metabolic cycle identified in [7] and [12] and the association we identify between growth rate and gene expression levels. This result is consistent with two possible explanations. The first is that there is variation in the growth rate of cells throughout the metabolic cycle. [12] and [32] have shown that under their specific experimental conditions, DNA replication and cell division is restricted to the reductive phases of the metabolic cycle. It is conceivable that growth per se (i.e. the accumulation of biomass) is paused during the reductive phases of the metabolic cycle so that the cell can replicate and segregate DNA and complete the complex processes of cell division; growth may then be restricted to the oxidative phase of the metabolic cycle. Alternatively, it is possible that as any heterogeneous culture grows faster, a greater fraction of cells are in the oxidative phase at any point in time. Thus, the growth rate gene expression signature we detect might reflect the fraction of cells in the oxidative and reductive phases of the metabolic cycle in a metabolically unsynchronized population.

The absence of growth rate differences during the cell division cycle (Figure 7B and 7C) supports our previous claim [4] that the growth rate expression signature is unrelated to the cell cycle. Moreover, since the published cell cycle experiments were performed in rich media using a fermentable carbon source, the results suggest that rapidly growing cells (which are almost exclusively fermenting) do not partition metabolic activity into discrete phases, as their energetic requirements are met in a continuously reductive metabolic state. It is only when slowed growth is imposed upon the cell, due to stress, nutrient limitation, or other suboptimal environments, that the metabolic cycle is required.

We sought to distinguish whether nutrient availability directly determines the transcriptional state related to growth rate or whether nutrient availability is integrated through an internal signaling pathway that controls the appropriate transcriptional state. To address this issue, we examined the regulatory circuit responsible for transcriptional changes in response to glucose availability in yeast. Glucose addition to cells growing on glycerol elicits a rapid and massive change in the pattern of gene expression, with more than half of all genes changing at least twofold in expression. Previous work has shown that the Ras/cAMP/PKA pathway is the major source for eliciting this transcriptional change in response to glucose addition [9],[11]. Activation of the Ras/PKA pathway in the absence of environmental signals, through induction of an activated allele of *RAS2* (*RAS2* ^G19V^), recapitulates in magnitude and direction more than 85% of the changes observed by glucose addition, and inhibition of PKA (concurrent with addition of glucose) blocks most of the glucose induced transcriptional changes ([11], Table S3). This mutation thereby represents a useful model connecting *S. cerevisiae*'s glucose sensory signaling to its transcriptional regulation of growth rate.

**Figure 8:**
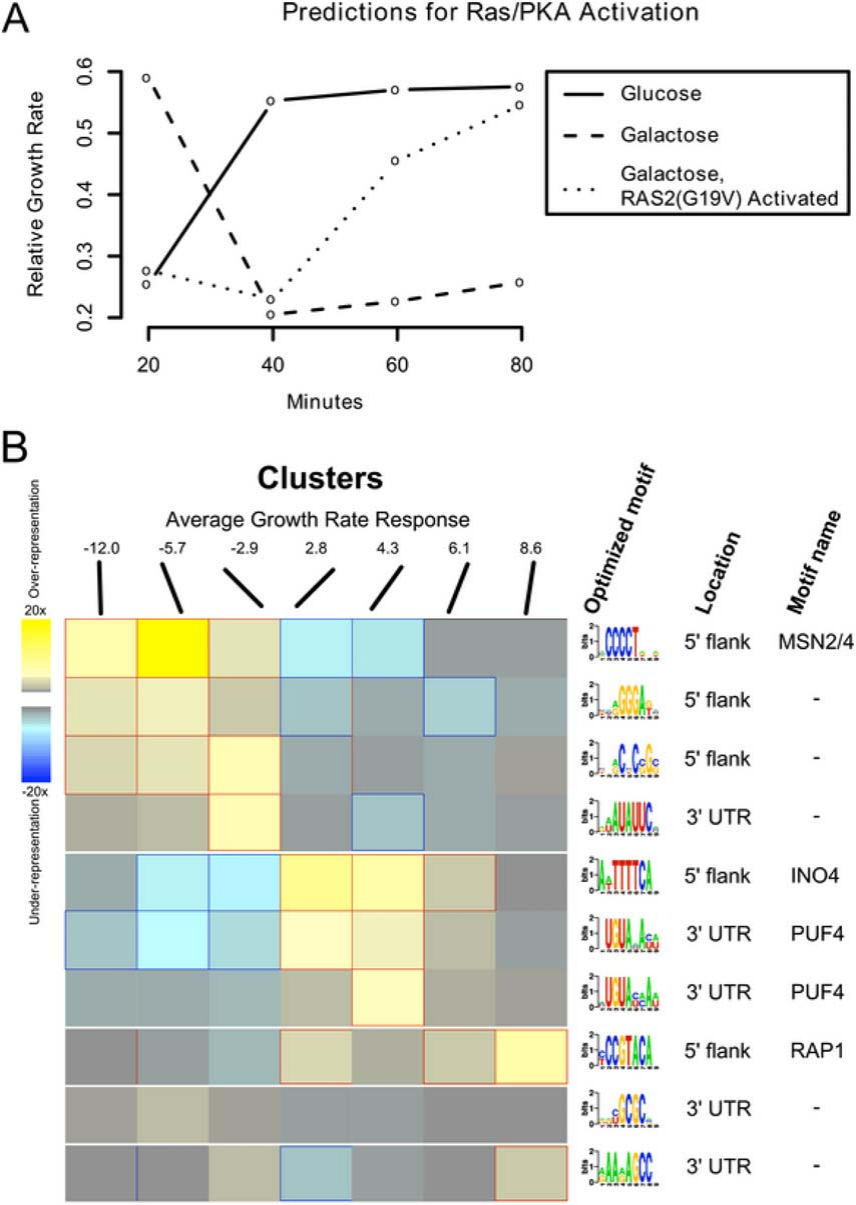
Perturbations and potential transcriptional regulators of the growth rate response. (A) Predicted growth rates for gal1Δ cells shifted to glucose, to galactose, and to galactose with a constitutively active RAS2G19V allele. On glucose, rapid growth is induced within ∼40 m; growth on galactose falls to low levels within ∼40 m, as it cannot be metabolized by this mutant. However, when glucose sensing is emulated by artificial activation of the Ras/PKA pathway, the transcriptional regulatory network attempts to induce rapid growth within ∼60–80 m despite the unavailability of appropriate nutrients. This disconnect between actual and perceived cellular state leads to cell death within 4–6 hours and suggests that nutrient sensing (as opposed to metabolic activity or internal cellular state) is responsible for a large portion of the transcriptional growth rate response. (B) Regulatory binding sites enriched in growth up- and down-regulated genes. We clustered the yeast genome by degree of growth rate response, yielding ten clusters with average responses ranging from −12.0 (strongly downregulated with increasing growth rate) to 8.6 (strongly upregulated). The FIRE program [34] predicted 10 regulatory motifs in the upstream flanks and 3′ UTRs of the most up- and down-regulated clusters. These included the known stress-responsive MSN2/4 binding sites in downregulated genes, the ribosomal regulators RAP1 and PUF4 in upregulated genes, and INO4 sites in upregulated genes (possibly corresponding to its role in the stress response and fatty acid biosynthesis [35]. We also identified five additional putative growth regulatory sites for which the binding factor is not yet known.

We used a *gal1*Δ strain carrying the activated allele *RAS2* ^G19V^ under control of the galactose inducible *GAL10* promoter. Addition of galactose activates the Ras/PKA pathway, but since galactose cannot be metabolized by this strain, the metabolic state of the cell remains unaltered [9]. When grown on glycerol our model predicts a relative growth rate of ∼0.2 for this strain (Figure 8A), which changes to ∼0.6 within twenty minutes following glucose addition, consistent with the change in doubling time from 5.8 hr to 2.6 hr. When we performed the same experiment on glycerol media and induced the *RAS2* ^G19V^ by means of galactose addition, we detected a transcriptional response within sixty minutes. The predicted growth rate of the *RAS2* ^G19V^ mutant strain was comparable to the addition of glucose despite the fact that galactose addition does not yield an increase in growth, as measured by optical density, since the cells are unable to metabolize galactose. In fact, while the model's summarization of gene expression state indicates that the culture is attempting to increase growth, induction of the *RAS2* ^G19V^ allele results in an immediate decrease in growth rate and complete cessation of growth within four hours [33]. These results are consistent with the cell setting its growth-specific transcription program on the basis of its *perception* of nutrients present in the environment, rather than on the direct availability of energy or metabolites produced from such nutrients. The mechanism by which the cell integrates this external state in order to set the appropriate growth rate expression state must be mediated, at least in part, through the Ras/cAMP/PKA pathway.

### Potential Transcriptional Regulators of Growth Rate

To investigate the regulatory basis of growth-associated gene expression, we identified motifs enriched in the 3′ and 5′ regions of genes with strong growth rate responses (Figure 8B). We assigned genes to clusters based on their growth rate response parameter (*β_g_*) using k-means clustering with k = 10. Using the FIRE motif identification program [34], we identified enriched motifs in seven of the resulting ten clusters. Consistent with the functional enrichments of negatively growth rate correlated genes [4], we identified known binding sites associated with the stress responsive transcription factors Msn2p and Msn4p in genes negatively correlated with growth rate. Conversely, genes that increase in expression with increased growth rate are enriched for the Rap1p consensus motif, which is commonly found upstream of genes encoding protein components of the ribosome.

We also found enrichment of the Ino4p binding site in genes upregulated with increasing growth rate. Ino4p forms a heterodimer with Ino2p to activate genes involved in phospholipid, fatty acid, and sterol biogenesis, all of which are required in greater abundance with increased growth rates. Furthermore, Ino4p has been proposed to have an inhibitory effect on a number of genes, including those that encode the heat shock proteins (Hsp12p, Hsp26p) and catalase (Ctt1p) [35]. We also identified two additional enriched motifs in the 5′ UTR for which the binding factor is not known, suggesting that additional activators of growth-related transcriptional programs remain to be determined.

In addition to 5′ upstream motifs, we identified five enriched 3′UTR motifs, which are potential binding site for proteins that promote mRNA degradation. Only a small number of mRNA binding consensus sequences are known in yeast, all of which belong to the Puf family of mRNA binding proteins [36]. Our analysis identified five enriched motifs in 3′UTRs. Two of these motifs, found in genes positively correlated with growth rate, were identified by the FIRE program as being targets of Puf4p. As an independent test, we compared the distribution of growth rate responses in the known gene targets of the five Puf proteins with the overall distribution of growth rate slopes. Targets of both Puf3p (220 genes) and Puf4p (205 genes) are enriched for genes that are upregulated with increasing growth (Wilcoxon-Mann-Whitney two sample p-values 9×10^−23^ and 7.23×10^−16^, respectively; Figure S3). The consensus motifs of Puf3p and Puf4p are very similar; investigation of the PUF4 motif identified by FIRE suggests that the enrichment signal for at least one of the motifs denoted PUF4 is likely to result from a composite of Puf3p and Puf4p target genes (Figure 8B).

Overall, this analysis is consistent with tight transcriptional regulation underlying the cellular growth program; it is likely that mRNAs involved in this process are also subject to extensive post-transcriptional control. Interestingly, since our growth-rate prediction method is sensitive to changes in gene expression levels that occur within minutes of a perturbation, we expect that post-transcriptional regulation (both mediated decay of and stabilization of transcripts) is involved in this response. Experimental analyses of the effects of perturbations within this regulatory network promise to shed further light on its organization.

## Discussion

We present a statistical model of the gene expression response to changes in growth rate in *S. cerevisiae*. Developed on expression levels from a variety of steady state growth rates and nutrient limitations, the model captures information regarding each gene's linear response to growth rate. As detailed in [4], approximately half of the genome shows a significant transcriptional response to growth rate with strong functional cohesiveness; here, we extend this model to show its robustness, applicability to new data, and ability to provide insight into the biological systems driving cellular regulation of growth rate. New experiments with more complex models (quadratic and hierarchical) demonstrated that additional model parameters did not provide substantial performance gains, in terms of growth rate prediction accuracy, particularly relative to their added complexity (data not shown). Similarly, variations in the definitions of responding genes or of growth-specific genes did not substantially alter results. This stability is reflected in the out-of-sample validation results, which quantify the model's accuracy in predicting relative growth rates from gene expression data, and in Table 2, which suggest that growth-specific signal is localized to a small number of genes consistently across experiments.

The model can be applied to new gene expression data to estimate the instantaneous growth rate of the originating cellular culture. The estimated instantaneous rate represents a measurement of the transcriptional state of cellular growth rate control, and it provides insight into the cell's growth rate at arbitrarily short time scales inaccessible by experimental measurements (e.g. optical density). Moreover, genes with unexpectedly high or low expression values can be detected during growth rate inference, and may indicate biological responses to non-growth stimuli. The predictions based on the proposed model are robust to changing biological conditions, experimental methods, and technological platforms; they also extend to the related yeast *S. bayanus* and the highly diverged yeast *S. pombe*, suggesting that the transcriptional control of growth rate captured by the model are a fundamental aspect of unicellular biology.

Through further analysis, we discovered several putative transcription factor binding sites enriched in growth-correlated genes, most notably the stress-responsive Msn2p and Msn4p, the Rap1p ribosomal factor, and Ino4p. Importantly, we have identified a likely role for post-transcriptional regulation in modulating transcriptional states related to growth rates. This finding is consistent with our ability to measure changes in growth rate over very short time scales using gene expression signatures. The abundance of any messenger RNA is a function of both its rate of production and of its rate of degradation; however, since transcription is relatively slow, changes in mRNA abundance can be most rapidly instantiated by altering the stability of the existent mRNA population. The Puf proteins have known roles in mediating mRNA degradation [37] and in mediating the association of functionally related transcripts [36]. It has recently been proposed that modulation of mRNA stability is an important factor in metabolic regulation [38]. The association of Puf protein binding domains in the 3′ UTRs of genes with increased expression at higher growth rates suggests that modulating mRNA stability is also important in the regulation of the growth response at short time scales.

From a statistical perspective, it is notable that a simple linear model accurately and robustly captures a specific biological phenomenon. The model represents a concise, functionally cohesive set of expression profiles regarding the genome's transcriptional response to growth. This functional interpretation of the model agrees with known aspects of the growth response, such as the transcription of ribosomal components, and provides insight as to the mechanistic roles of internal feedback, environmental sensing, and the stress response as growth rate varies. By monitoring a small ensemble of genes—with few parameters per individual gene—the model is easily applicable to new conditions and organisms and is robust to technical and biological sources of variation. These features enable our model to serve both as a practical tool for growth rate estimation (available at http://function.princeton.edu/growthrate) and as a mechanistic building block in the pursuit of a systems-level understanding of cellular growth processes.

## Supporting Information

Dataset S1 An RData archive containing the complete collection of programs and results. The archive includes a Table (named frmeGRParameters) with the growth rate slope, goodness of fit, and other parameters based on our expression data and linear model. The linear model assigns each gene a growth rate slope (i.e. response to increased growth rate), baseline response, and goodness of fit (i.e. linearity of response) based on our 36 expression arrays. The statistical significance of these parameters was tested against a null distribution based on 100,000 bootstrap samples. We have also indicated whether each gene is in our positively or negatively growth correlated gene sets, whether it is up- or down-regulated in the Environmental Stress Response (ESR) [6], whether it was used as a growth-specific gene for inferring instantaneous growth rates, and whether it was reliably unresponsive to changes in growth rate.
(10.88 MB ZIP)

Figure S1 Growth rate predictions for chemostat cultures subjected to a brief heat pulse at various flow rates. Expression time courses were taken for a collection of chemostats at increasing growth rates, each subjected to a brief (<30 s) heat pulse at time zero; see Supplemental Table S1 for details. Predicted growth rates show an immediate departure from steady state as the heat pulse is administered immediately before time zero, followed by a gradual return to steady state and regulatory overshoot. This behavior is consistent across growth rates, with the lowest growth rates potentially showing a lesser shock response due to stress tolerance.
(0.02 MB PDF)

Figure S2 Growth rate predictions for all conditions in the stress response expression arrays in [6]. These predictions are generally consistent with known yeast biology and agree with expected growth behavior; most shock time courses, including all heat shocks, peroxide, diamide, and hyper-osmotic stress, provoke an initial sharp decrease in growth rate followed by a return to initial or near-initial rate. Shorter shocks, such as DTT, menadione, and peroxide responses, capture only the rate decrease. Batch growth proceeds at a fairly constant rate until nutrients become depleted, at which point the rate decreases sharply; this pattern is also seen in intentional nitrogen depletion. Growth rates across varying temperatures peak as expected at 25 C, falling off at lower and higher temperatures. Response to varying carbon sources is also as expected, with ethanol inducing the slowest growth and fructose, sucrose, and glucose allowing the most rapid. The model's inference of growth rate from expression data alone thus allows both post hoc growth analysis (e.g. years after the original experiment) and an estimation of growth rates for cultures where it would be difficult or time consuming to measure directly.
(0.03 MB PDF)

Figure S3 PUF3 and PUF4 targets are enriched for genes that respond positively to growth. We plotted the distribution of PUF3 targets (220 genes; black line) and PUF4 targets (205 genes; red line) identified in [36] on the distribution of slopes reported in [4]. Targets of both these mRNA-binding proteins are enriched for genes that are increased in expression at higher growth rates. This is consistent with an important role for post-transcriptional regulation in modulating the growth-related gene expression program.
(0.05 MB PDF)

Table S1 Expression of growth-specific genes for chemostat cultures at increasing growth rates exposed to a brief heat pulse. A collection of chemostats was run at growth rates ranging from 0.05/hr to 0.25/hr. A brief (<30 s) heat pulse was administered immediately before time zero, and expression arrays were collected in a time course from before the pulse (pre.) to two hours after the pulse using the 0.1/hr pre-pulse time point as a reference. (Here we provide expression data for all the growth-specific genes. The genome-wide collection of gene expression data will appear in a subsequent publication.)
(0.06 MB XLS)

Table S2 Expression of growth-specific genes for chemostat cultures at increasing growth rates limited on various nitrogen sources. A collection of chemostats was run at growth rates from ∼0.06/hr to ∼0.21/hr limited on one of several different nitrogen sources, including ammonium, allantoin, glutamate, arginine, glutamine, urea, and proline. (Here we provide expression data for all the growth-specific genes. The genome-wide collection of gene expression data will appear in a subsequent publication.)
(0.04 MB XLS)

Table S3 Expression of growth-specific genes for batch cultures grown on glucose, galactose, and galactose with a constitutively activated Ras/PKA pathway. We constructed a gal1 deletion strain carrying the activated allele RAS2(G19V) under control of the galactose inducible GAL10 promoter. Addition of galactose activates the Ras/PKA pathway, but since galactose cannot be metabolized by this strain, the metabolic state of the cell remains unaltered. Gene expression was then assayed at 20, 40, 60, and 80 minutes (relative to time 0) after nutrient exposure. (Here we provide expression data for all the growth-specific genes. See [9] for additional data.)
(0.03 MB XLS)

Table S4 Expression of growth-specific genes for Saccharomyces bayanus orthologs under the diauxic shift and heat shock. Gene expression was measured for time courses of S. bayanus undergoing the diauxic shift and for a culture heat shocked by shifting from 25 to 37 C. (Here we provide expression data for all the growth-specific genes. The genome-wide collection of gene expression data will appear in a subsequent publication.)
(0.03 MB XLS)

Table S5 S. cerevisiae growth-specific genes used for growth rate prediction in this study with S. bayanus and S. pombe orthologs. S. cerevisiae growth-specific genes were defined to have a bootstrapped p-value of growth rate response and linear fit less than 10−5. S. bayanus orthologs were drawn from [19] and S. pombe orthologs from [20].
(0.00 MB XLS)

Table S6 Predicted relative growth rates for expression data from the deletion collection in [10]. Our predictions for the 199 mutants for which Hughes et al directly measured growth rates show significant correlation to the experimental gold standard (rho = 0.473, p<10−11), in contrast to other single mutant fitness estimates based on growth curve analysis (e.g. [23] reports rho = 0.321, p<10−6; [24] reports rho = 0.108, p>0.2).
(0.01 MB XLS)
