# Supplementary material for: Predicting Cellular Growth from Gene Expression Signatures
Source: PLoS Comput Biol. 2009 Jan 2;5(1):e1000257. doi: 10.1371/journal.pcbi.1000257 (PMC2599889; doi:10.1371/journal.pcbi.1000257)

Predictions for Chemostat Heat Pulse, 0.05/hr

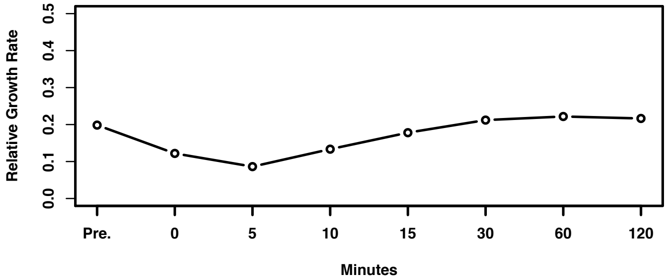

Predictions for Chemostat Heat Pulse, 0.1/hr

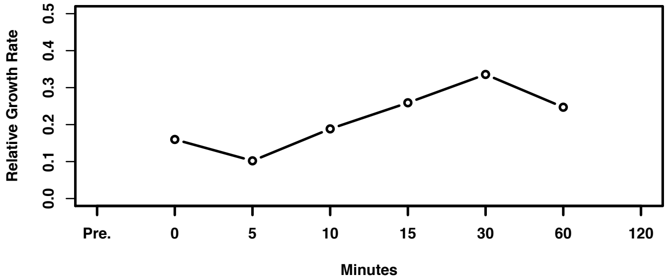

Predictions for Chemostat Heat Pulse, 0.15/hr

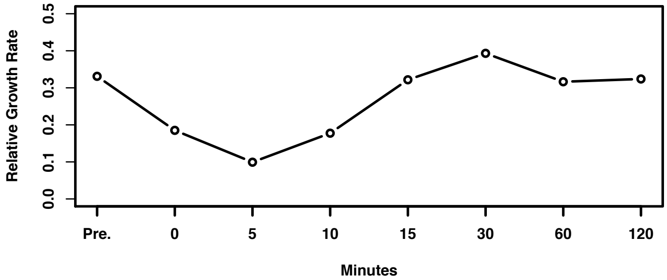

Predictions for Chemostat Heat Pulse, 0.2/hr

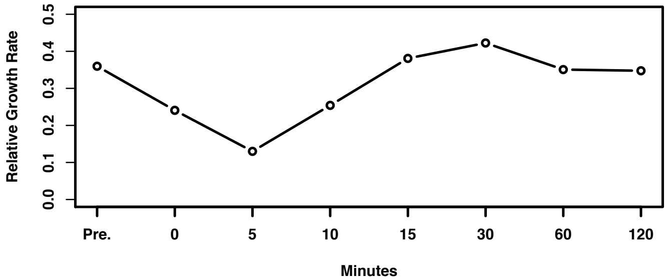

Predictions for Chemostat Heat Pulse, 0.25/hr

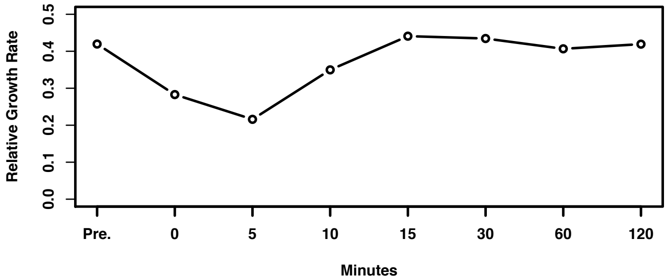

Supplement: Figure S1 — Growth rate predictions for chemostat cultures subjected to a brief heat pulse at various flow rates. Expression time courses were taken for a collection of chemostats at increasing growth rates, each subjected to a brief (<30 s) heat pulse at time zero; see Supplemental Table S1 for details. Predicted growth rates show an immediate departure from steady state as the heat pulse is administered immediately before time zero, followed by a gradual return to steady state and regulatory overshoot. This behavior is consistent across growth rates, with the lowest growth rates potentially showing a lesser shock response due to stress tolerance. (0.02 MB PDF) [file pcbi.1000257.s002.pdf]
