# Supplementary material for: Predicting Cellular Growth from Gene Expression Signatures
Source: PLoS Comput Biol. 2009 Jan 2;5(1):e1000257. doi: 10.1371/journal.pcbi.1000257 (PMC2599889; doi:10.1371/journal.pcbi.1000257)

Predictions for Gasch 2000 Adenine Starvation

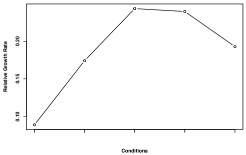

Predictions for Gasch 2000 Carbon Sources

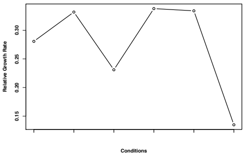

Predictions for Gasch 2000 Diamide

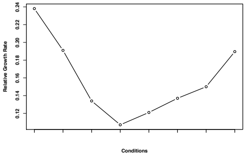

Predictions for Gasch 2000 DTT Time Course

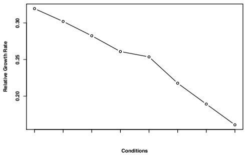

Predictions for Gasch 2000 DTT Pool Time Course

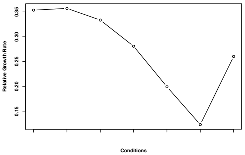

Predictions for Gasch 2000 HO Response

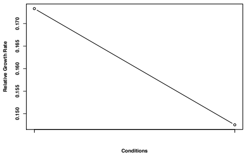

Predictions for Gasch 2000 HO Time Course

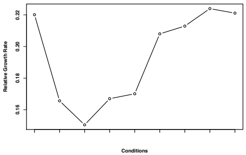

Predictions for Gasch 2000 Heat Shock, 25–37C

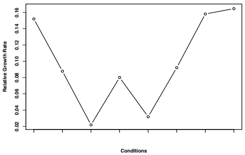

Predictions for Gasch 2000 Heat Shock, 29–33C

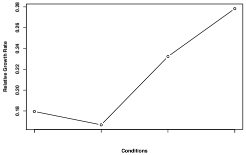

Predictions for Gasch 2000 Heat Shock, 30–37C

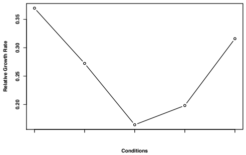

Predictions for Gasch 2000 Heat Shock, 37–25C

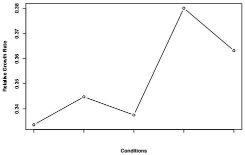

Predictions for Gasch 2000 Heat Shock, Mild

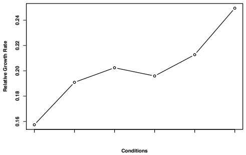

Predictions for Gasch 2000 Heat Shock to 37C

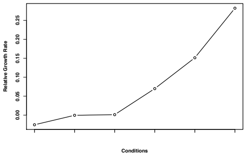

Predictions for Gasch 2000 Hyper–osmotic Shock

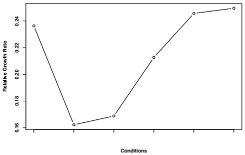

Predictions for Gasch 2000 Hypo–osmotic Shock

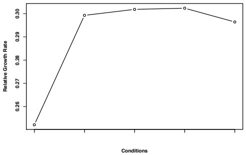

Predictions for Gasch 2000 Menadione

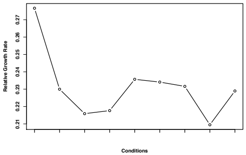

Predictions for Gasch 2000 Nitrogen Depletion

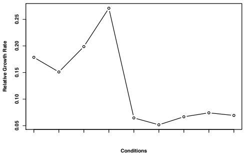

Predictions for Gasch 2000 Stationary Phase, 25C Time Course

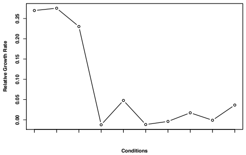

Predictions for Gasch 2000 Stationary Phase, 30C Time Course

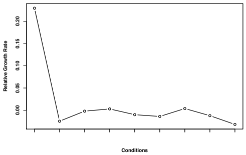

Predictions for Gasch 2000 Steady State, Inc. Temp.

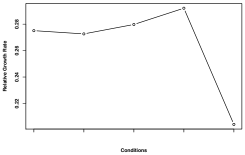

Predictions for Gasch 2000 Steady State, Pool Inc. Temp.

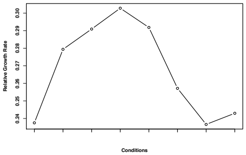

Supplement: Figure S2 — Growth rate predictions for all conditions in the stress response expression arrays in [6]. These predictions are generally consistent with known yeast biology and agree with expected growth behavior; most shock time courses, including all heat shocks, peroxide, diamide, and hyper-osmotic stress, provoke an initial sharp decrease in growth rate followed by a return to initial or near-initial rate. Shorter shocks, such as DTT, menadione, and peroxide responses, capture only the rate decrease. Batch growth proceeds at a fairly constant rate until nutrients become depleted, at which point the rate decreases sharply; this pattern is also seen in intentional nitrogen depletion. Growth rates across varying temperatures peak as expected at 25 C, falling off at lower and higher temperatures. Response to varying carbon sources is also as expected, with ethanol inducing the slowest growth and fructose, sucrose, and glucose allowing the most rapid. The model's inference of growth rate from expression data alone thus allows both post hoc growth analysis (e.g. years after the original experiment) and an estimation of growth rates for cultures where it would be difficult or time consuming to measure directly. (0.03 MB PDF) [file pcbi.1000257.s003.pdf]
