# Supplementary figures and images for: Predicting Cellular Growth from Gene Expression Signatures
Source: PLoS Comput Biol. 2009 Jan 2;5(1):e1000257. doi: 10.1371/journal.pcbi.1000257 (PMC2599889; doi:10.1371/journal.pcbi.1000257)

## Distribution of Slopes

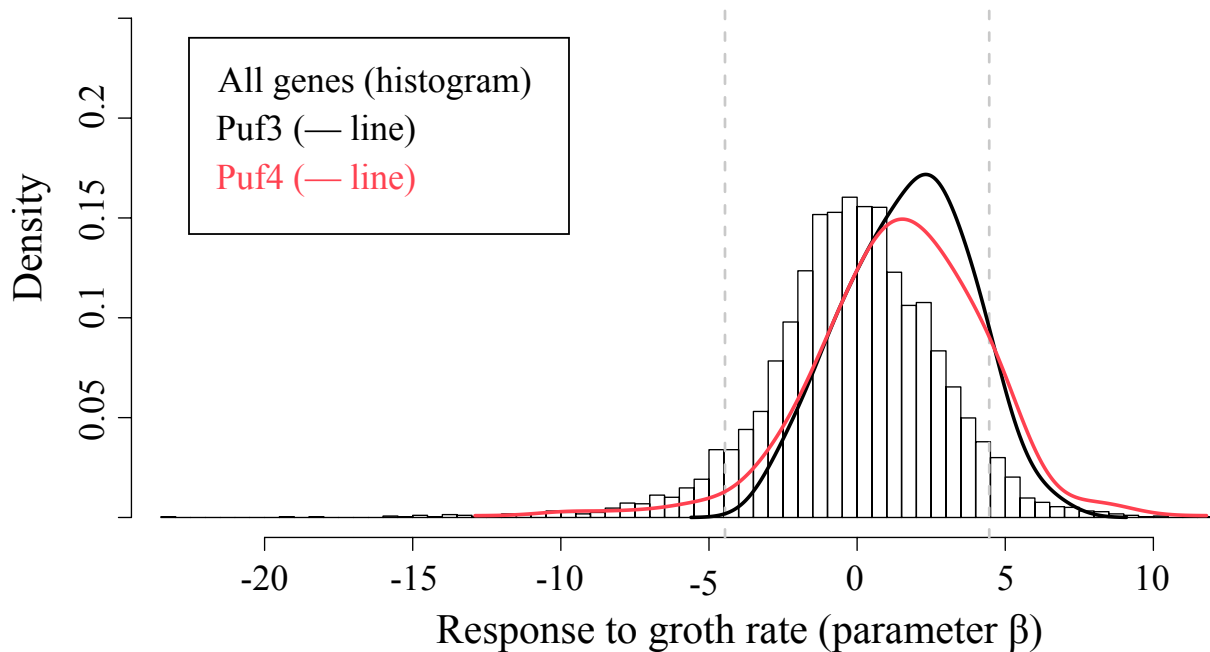

Supplement: Figure S3 — PUF3 and PUF4 targets are enriched for genes that respond positively to growth. We plotted the distribution of PUF3 targets (220 genes; black line) and PUF4 targets (205 genes; red line) identified in [36] on the distribution of slopes reported in [4]. Targets of both these mRNA-binding proteins are enriched for genes that are increased in expression at higher growth rates. This is consistent with an important role for post-transcriptional regulation in modulating the growth-related gene expression program. (0.05 MB PDF) [file pcbi.1000257.s004.pdf]
